# Illness perceptions and health outcomes among community-dwelling older adults with multiple chronic conditions

**DOI:** 10.1093/geroni/igab046.2344

**Published:** 2021-12-17

**Authors:** Ayomide Bankole

**Affiliations:** University of North Carolina at Chapel Hill, Chapel Hill, North Carolina, United States

## Abstract

Illness perceptions (IP) has been associated with self-management and health outcomes in individuals with chronic diseases such as heart disease and diabetes; however, there is less research on the relationship between IP and health outcomes in individuals with multiple chronic conditions (MCC). Older adults with MCC are more likely to experience poor outcomes such as hospitalizations and poor self-rated health yet, there is less understanding of the processes associated with these outcomes. The purpose of this study was to (1) explore the relationship between IP and self-rated health among older adults with MCC (2) explore the relationship between IP and the number of hospitalization within the past year among older adults with MCC. Understanding these relationships may be instrumental to designing targeted interventions to improve health outcomes for this population. 116 participants (ages 65-90) completed the illness perception of multimorbidity scale, modified general health subscale of the SF-36 questionnaire, and self-reported number of hospitalizations within the past year. Ordinal logistic regression was used for analysis. Older adults who reported negative IP were likely to report worse self-rated health and this relationship remained significant after controlling for age and number of chronic conditions {-0.032 (95% CI (-0.050 to 0.014) p< 0.05}. There was no significant relationship between IP and the number of hospitalization within the past year. The study results study suggest that IP is associated with self-rated health in older adults with MCC. IP may be useful to design targeted interventions to improve self-rated health in this population.

